# Anecdotical

**Published:** 1855-09

**Authors:** 


*Anecdotical.*—Sir Richard Jebb used to tell a story of himself which made even avarice comical. Attending a nobleman whom he thought ought to pay him five guineas at each visit, he received only three. Suspecting some trick on the part of the steward, from whom he received it, he, at the next visit, contrived to drop the three guineas. They were picked up and again deposited in his hand; but he still continued to look on the carpet. His lordship asked if all the guineas were found. "There must be two still on the floor," replied Sir Richard, "for I have but three." The hint was taken, and his trick was successful.

*Prof. Chapman.*—Harper's Magazine has gathered some of the best of the witticisms of Chapman, but we do not recollect to have seen in print any report of the conversation between him and the veteran Dr. Francis, of New York. Dr. F. was saying that maternal serpents would swallow their young on the approach of danger, and stated that he had himself seen a brood of seven infantile black snakes disappear down the throat of the mother for protection, when alarmed.

"I think," said Chapman, "that that is a very large dose of Serpentaria!"

"Oh no!" said Mr. Bogg, "Our friend can't live long, poor fellow! His constitution is all gone."

"But if his constitution is all gone, then how does he live at all?"

"Oh! ah! yes! ahem! *he is living on the by-laws!*"

We have often thought of some very sick people that they ought to be arrested under the vagrant act, for living "without visible means of support."

